# Severe Loss of Appetite in Amyotrophic Lateral Sclerosis Patients: Online Self-Assessment Study

**DOI:** 10.2196/ijmr.2463

**Published:** 2013-04-17

**Authors:** Teresa Holm, André Maier, Paul Wicks, Dirk Lang, Peter Linke, Christoph Münch, Laura Steinfurth, Robert Meyer, Thomas Meyer

**Affiliations:** ^1^Department of Neurology, CharitéUniversity HospitalBerlinGermany; ^2^PatientsLikeMeLondonUnited Kingdom; ^3^Department of Psychiatry and Psychotherapy IIIUniversity Hospital UlmUlmGermany

**Keywords:** amyotrophic lateral sclerosis, nutrition, loss of appetite, weight loss, online self-assessment

## Abstract

**Background:**

Undesirable loss of weight is a major challenge in amyotrophic lateral sclerosis (ALS). However, little is known about loss of appetite in ALS patients.

**Objective:**

We investigated loss of appetite in ALS patients by means of an online self-assessment and whether ALS-related symptoms were associated with it.

**Methods:**

Loss of appetite in 51 ALS patients was assessed using the Council on Nutrition Appetite Questionnaire (CNAQ). Loss of appetite is defined as a CNAQ-score of 28 or less with a predicted weight loss of at least 5% within 6 months. We developed an Internet portal to facilitate self-assessment.

**Results:**

Approximately half of the ALS patients (47%, 24/51) suffered from severe loss of appetite; after 6 months this increased to nearly two-thirds (65%, 22/34). An average weight loss of 5% was found in the group with severe loss of appetite as compared to only 2% of patients with normal appetite. Interestingly, loss of appetite was associated with respiratory dysfunction (*P*=.001, R^2^=.223).

**Conclusions:**

Loss of appetite was more common and more severe than expected. It was found to be an independent risk factor for unintended weight loss and may be related to dyspnea. The impact of severe loss of appetite on survival and quality of life should be established in further studies.

## Introduction

Amyotrophic lateral sclerosis (ALS) is a neurodegenerative disease resulting from the progressive degeneration of upper and lower motor neurons of the spinal cord, the brainstem and the cerebral cortex.

In the course of the disease, 15-55% of patients suffer from clinically severe weight loss [1-4]. Nutritional status is an important prognostic factor for survival in ALS [5-8]; weight loss that leads to a body mass index (BMI) below 18.5 kg/m^2^ results in a 7.7 times higher mortality rate, compared to patients with normal weight [5]. The underlying causes of weight loss associated with ALS are heterogeneous [1,6] but are likely to include malnutrition, hypermetabolism, cachexia, and loss of appetite [9-11]. Loss of appetite is a multifactorial syndrome resulting from a number of symptoms such as changes in controlling eating behavior, depression, and psychological distress [12].

The revised ALS Functional Rating Scale (ALSFRS-R) is an established and internationally used self-assessment questionnaire measuring physical functions of ALS patients in activities of daily living [13]. Based on its simplicity and ability to reflect disease progression, the ALSFRS-R is routinely applied in most clinical trials and in clinical practice. The instrument primarily focuses on the functional impact of muscle weakness, and does not attempt to capture important symptoms such as loss of appetite.

In the clinical setting, ALS patients reported regularly from changes in presenting appetite associated with a decline in caloric intake (with a reduction of the portion size) during the course of the disease. The aim of the present study was to determine the frequency of loss of appetite in ALS patients. This investigation does not claim to validate the Council on Nutrition of Appetite Questionnaire (CNAQ) in ALS. We used an online patient portal to field the CNAQ—a patient reported outcome that records loss of appetite [14]. CNAQ was developed as a short, simple appetite assessment tool in long-term care in institutionalized and community-dwelling adults. The CNAQ has not been deployed in ALS before. Within our study population, we grouped patients according to their ALS-related symptoms to identify risk factors that would be associated with decrease in appetite. We hypothesized that loss of appetite might be associated with dyspnea or dysphagia which are common symptoms in ALS.

## Methods

### Overview

Between April and November 2010, 51 patients were consecutively recruited at the Department of Neurology at the Charité University Hospital of Berlin. Patients gave written informed consent for their participation. Patients with possible, probable, or definite ALS (according to the revised El Escorial Criteria [15]) were enrolled in the study. Exclusion criteria included lack of Internet access in the patients’ environment, patients suffering from consumptive disease or from eating disorder, and patients with enteral feeding. However, patients without Internet connection were able to participate in the trial if the next caregivers provided an alternative Internet access for the online self-assessment. Also patients presenting clinical criteria for cognitive impairment, especially frontotemporal dementia, were not included. These symptoms however, were not explicitly tested. Patients underwent neurological examination and measurements for slow respiratory vital capacity, height, and body weight were taken throughout from ALS outpatients. The ALSFRS-R was obtained during Web visits for monitoring the individual disease progression.

### Nutritional Assessment

BMI was calculated by using the formula BMI = weight (kg) / height (m)^2^. Malnutrition was defined by a BMI less than 18.5 kg/m^2^ in ALS patients up to the age of 65 years, a BMI of <20 kg/m^2^ in patients over 65 years [2,5], severe weight loss of 3.5% in 3 months, 5% in 6 months, or 10% in 1 year [2,5,16].

### Appetite Assessment

The CNAQ was used for measuring loss of appetite. This assessment tool has not been specifically developed and validated for ALS. The CNAQ contains 8 single domain items, each rated on a 5-point scale. Thus, the total score can range between a minimum of 8 and a maximum of 40 points. While lower scores indicate deterioration in appetite, a total score of 28 or less is defined as “severe loss of appetite” and predicts a weight loss of at least 5% within the next 6 months [14]. This prospective questionnaire was developed as a short, simple appetite assessment tool for patients in long-term care in institutionalized and community-dwelling adults. Given the lack of appetite-related sub-scores within the established ALS rating scales, we decided to use the CNAQ, which does not include any motor symptom related items. Therefore, the CNAQ is unlikely to directly reflect difficulty in chewing and swallowing or the motor disability of patients to care for themselves. The CNAQ score contains one question concerning the mood of the patient. Although this item contributes to loss of appetite, it may interfere with other ALS related symptoms since anxiety and depression occasionally occur in ALS.

### Online Self-Assessment

In the course of ALS, patients need alternative ways of communicating, especially because of dysarthria and progressing physical impairment. An increasing number of patients rely on novel methods, such as the Internet, for communication; therefore we chose the Internet self-assessment method for completion of questionnaires. The Internet portal ALShome was created as a safe Web application for collecting patient-related data and has been described previously [17]. Patients had controlled access to this website using an automatically generated username and password. Participants were asked to perform online self-assessments once a week over a period of 6 months. The study period was based on the ability of the CNAQ to predict weight loss after 6 months. We used monthly average CNAQ scores for data analysis.

Approval was obtained from the ethical review committee and Data Security Officer from the Ethikkommission der Charité, Universitätsmedizin Berlin, for online self-assessment.

### Classification of Patients

The study population was clustered by the occurrence of ALS-associated symptoms. Functional impairment was assessed by the ALSFRS-R; the score contains 12 items, each scored from 0 to 4. According to our hypothesis, we clustered patients into 2 groups based on the following 4 categories within the ALSFRS-R: (1) swallowing impairment (mild to severe vs without), (2) dyspnea (mild to severe vs without), (3) orthopnea (mild to severe vs without), and respiratory insufficiency (using non-invasive ventilation, NIV, vs without NIV). Patients scoring between 0 to 3 points on each single ALSFRS-R item displayed mild to severe physical impairment and were thus classified as ‘”mild to severe”, while patients scoring 4 points were classified as “not functionally affected”.  Within the group of patients suffering from mild to severe swallowing difficulties, individuals with percutaneous endoscopic gastrostomy (PEG) were excluded because the CNAQ was, by definition, not applicable in these patients [14].

### Data Analysis

Relevant data was recorded via the Web-based database and analyzed with PASW Statistics version 19.0 for Windows. Regarding the CNAQ independent two-sample *t* tests with between subject factor group (patients with “mild to severe” symptoms vs patients “not affected”) and within subject factor symptoms (swallowing impairment, dyspnea, orthopnea, and respiratory insufficiency) were performed at baseline. For further analysis a multiple linear regression was applied. For analyzing the BMI data and mean CNAQ scores (baseline vs follow-up) we used the dependent *t* test for paired samples. The significance level was tested using a two-tailed test at *P*=.05. Mean values and SD are given.

## Results

A total of 51 patients were enrolled in this study, including 34 males with the mean age of 58.4 (SD 9.4, range 37-73) years and 17 females with the mean average age 59.1 (SD 7.7, range 42-73) years. The mean disease duration was 31.7 (SD 24.9, range 3-125) months. We included patients with spinal (36/51, 71%), bulbar (13/51, 26%), and axial (2/51, 4%) onset. The baseline characteristics of the 51 patients including neurological, nutritional, and respiratory examination status are presented in Table 1.

**Figure 1:**
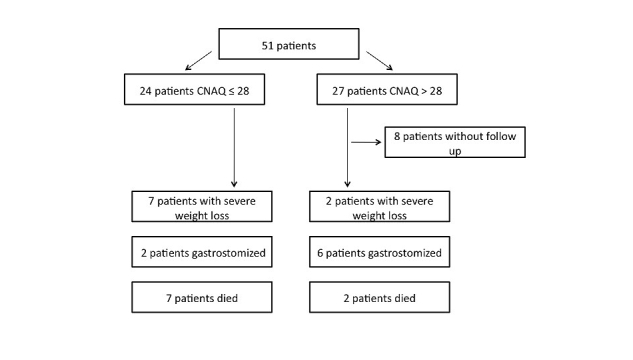
Flowchart of appetite assessment and main results after 6 months.

During the study period of 6 months, 8 patients underwent PEG. 9 patients died within the observation period. The majority of patients followed the study protocol including self-assessment throughout the 6 months of observation. Because of missing compliance and/or uncertain clinical course, 8 patients terminated the self-assessment prematurely. At baseline, assessment of appetite using the CNAQ revealed a severe loss of appetite (CNAQ≤28) in 47% (24/51) of the participants. The mean CNAQ score was 28.1 (SD 3.9, range 20-33). Participant flow is shown in Figure 1.

**Figure 2:**
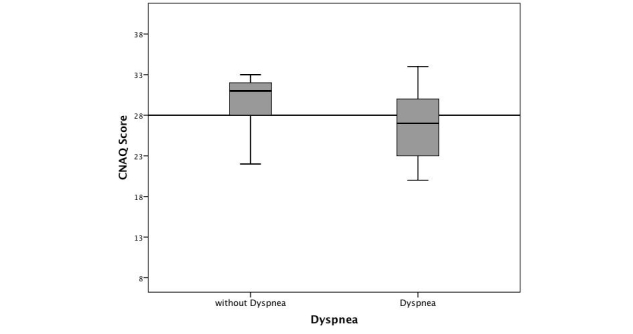
Box plots of CNAQ scores in relation to accordance of dyspnea.

Severe loss of appetite (CNAQ≤28) was identified in 59% (17/29) of patients suffering from mild to severe dyspnea (29/51), in contrast to only 32% (7/22) of patients without dyspnea (22/51; *t*_49_ = 2.610, *P*=.012, Table 2 and Figure 2).

The multiple linear regression analysis revealed that dyspnea (*P*=.001, *R* ^2^=.223) and age (*P*=.038, *R* ^2^=.223) were significantly correlated with loss of appetite. A similar (though non-significant) trend was found for orthopnea. Among 17 patients with mild to severe orthopnea, 59% (10/17) suffered from loss of appetite, compared to 41 % (14/34) of patients without orthopnea (*t*_49_=1.974, *P*=.060). 12 of our 51 patients were treated with NIV. Fewer NIV-treated patients (5/12, 42%) had severe loss of appetite than patients not treated with NIV (19/39, 49%). However, due to small sample numbers, these results should be interpreted with caution and further study is warranted.

Surprisingly, there was no significant difference on mean CNAQ score within the ALSFRS-R item, swallowing impairment (see Table 2).

**Table 1:** Descriptive characteristics of the study population during baseline visit. Numbers show mean, SD, and range.

Characteristic	Total n (%) or mean (SD, range)	Male n (%) or mean (SD, range)	Female n (%) or mean (SD, range)
n (%)	51 (100)	34 (67)	17 (33)
Age at onset in years, mean (SD, range)	56.3 (9.2, 36-72)	55.7 (9.5, 36-71)	57.3 (8.5, 38-72)
Duration of disease (months), mean (SD, range)	31.7 (24.9, 3-125)	31.0 (25.6, 3-125)	32.9 (24.2, 9-104)
ALSFRS-R score, mean (SD, range)	33.0 (8.1, 16-47)	32.5 (7.5, 19-47)	34.2 (9.3, 16-44)
Weight (kg), mean (SD, range)	72.5 (14.3, 42-105)	77.9 (13.3, 57-105)	61.8 (9.7, 42-84)
BMI (kg/m^2^), mean (SD, range)	23.6 (3.5, 17-32)	24.2 (3.5, 19-32)	22.4 (3.2, 17-29)
Vital capacity, % mean (SD, range)	65.6 (25.4, 14-107)	60 (25.6, 14-107)	75.5 (22.6, 23-107)
Spinal onset, n (%)	36 (71)	26 (77)	10 (59)
Bulbar onset, n (%)	13 (26)	6 (18)	7 (41)
Axial onset, n (%)	2 (4)	2 (6)	0 (0)
NIV, n (%)	12 (24)	9 (27)	3 (18)

**Table 2:** Descriptive characteristics of the study population during baseline visit and after 6 months divided into CNAQ scores (CNAQ ≤ 28 and CNAQ >28). Numbers show mean, SD, and range.

Characteristics	CNAQ≤28 n (%) or mean (SD, range)	CNAQ>28 n (%) or mean (SD, range)
Female: Male	7:17	10:17
Age at onset, mean (SD, range)	57.8 (10,0, 36-72)	54.4 (8.1, 37-69)
Duration of disease (months), mean (SD, range)	27.7 (23.8, 3-125)	35.1 (25.8, 4-104)
ALSFRS-R score at baseline, mean (SD, range)	33.1 (8.0, 16-47)	33 (8., 16-44)
ALSFRS-R score after 6 months, mean (SD, range)	25.9 (8.5, 15-40)	30.6 (9.0, 17-44)
BMI (kg/m^2^) at baseline, mean (SD, range)	23.1 (3.5, 19-32)	24.1 (3.4, 17-32)
BMI (kg/m^2^) after 6 months, mean (SD, range)	21.6 (3.3, 17-29)	23.2 (3.7, 18-30)
Vital Capacity at baseline, % mean (SD, range)	64.8 (23.2, 24-103)	70.7 (26.4, 23-107)
Spinal onset, n (%)	20 (83)	16 (59)
Bulbar onset, n (%)	3 (13)	10 (37)
Axial onset, n (%)	1 (4)	1 (4)
NIV, n (%)	4 (17)	8 (30)
Deceased, n (%)	7 (29)	2 (7)

At baseline, malnutrition was diagnosed in 46% (26/51) of the total study population [2,5,16]. 12% (7/51) had an abnormally low BMI and 40 % (23/51) had suffered from severe weight loss in the time leading up to baseline as defined in the methods section.

**Figure 3:**
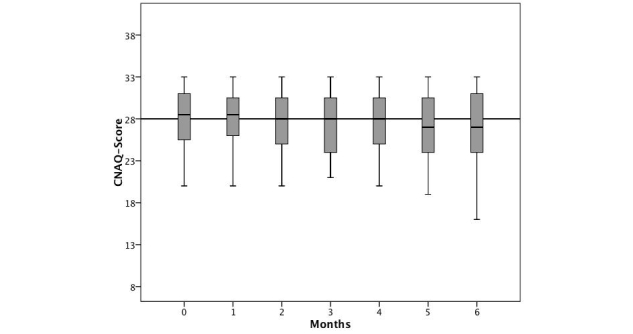
Box plots of CNAQ scores in the course of 6 months; patients receiving PEG (n=8) were excluded in the follow-up.

Loss of appetite worsened over time, with the average value of the CNAQ (mean 28.1, n=51 at baseline) decreasing to a mean of 26.5 (n=31) after 6 months (*t*_*30*_= 3.433, *P*=.002, Figure 3).

**Figure 4:**
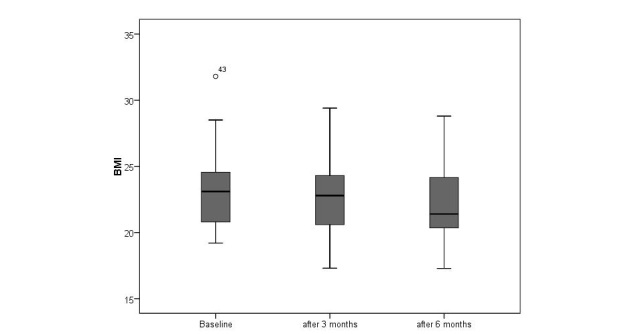
Changes in body weight over the course of 6 months in ALS patients suffering from severe loss of appetite (CNAQ ≤ 28).

**Figure 5:**
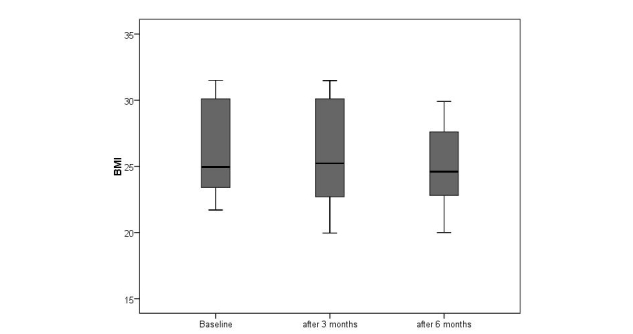
Changes in body weight over the course of 6 months in ALS patients with a CNAQ score > 28.

At baseline, severe loss of appetite was detected in 47% (24/51) of the patients; after 6 months this increased to 65% (22/34). During the observation period of 6 months, loss of appetite (CNAQ≤28) was associated with weight loss. The mean BMI in the severe loss of appetite group decreased significantly from 22.9 to 21.6 kg/m^2^ (*t*_*15*_=3.829, *P*=.002); a significant reduction was also found in the group without loss of appetite (CNAQ>28) with BMI reducing from 24.4 to 23.4 kg/m^2^ (*t*_*17*_=3.055, *P*=.007). However, the high degree of dysphagia in patients may have accounted for changes in the second group (ie, necessitating PEG within the study period). Repeating the analysis only in patients without high degree of dysphagia, the mean BMI in the loss of appetite group (CNAQ≤28) decreased from 23 to 21.8 kg/m^2^ (*t*_*14*_=3.467, *P*=.004; Figure 4), whereas in patients without loss of appetite (CNAQ>28; Figure 5) and no severe dysphagia, there was no significant weight loss (BMI 25.0 kg/m^2^ at baseline, 24.4 kg/m^2^ at follow-up; *t*_*12*_=1.961, *P*=.073).

In conclusion, after correcting for high degree of dysphagia, an average weight loss of 5% occurred after 6 months in the group of patients with a severe loss of appetite (CNAQ≤28), compared to 2% of weight loss in patients with a CNAQ score greater than 28. Additionally, in 24 patients presenting severe loss of appetite at baseline, 7 patients died during the observation period. In contrast, 2 patients of 27, who rated their CNAQ scores higher than 28 at baseline, died.

## Discussion

### Principal Findings and Conclusions

Appetite is defined as a pleasurable sensation or a desire to eat. For the first time we wanted to measure this feeling in the course of ALS, as it is an important part of quality of life especially in chronic diseases. There have been an increasing number of ALS patients reporting lack of appetite leading to reduced food intake during medical care. Using a combination of clinical examination and online self-assessment, about half of the study population showed severe loss of appetite, defined by a CNAQ score of 28 or less. During the course of the disease, both the prevalence and severity of appetite loss worsened. Our findings contributed to the notion that reduced appetite is a common ALS-associated symptom which may impair the individual capacity to maintain adequate nutrition. Previous reports have estimated weight loss exceeding 15-25% of body weight [3,4]. In fact, malnutrition is one of the most common symptoms and occurs in up to 50% of ALS patients [2]. Our finding of frequent loss of appetite in ALS appears to be in line with the previously reported malnutrition studies in ALS. Earlier work on nutritional status in ALS examined indicators of malnutrition such as weight loss, muscle wasting, body composition, and energy expenditure, but appetite has received little attention. Reduced appetite is a multifactorial syndrome due to changes in physiological eating behavior, but is also reinforced by depression [12]. Reportedly, about 10-20% of ALS patients suffer from depression [18-23]. Although potentially relevant, it is unlikely that depression alone explains the high prevalence of severe loss of appetite. However, it would be useful for future studies to assess depression in ALS to clarify its association with appetite. To our surprise, we did not found a significant correlation of appetite to dysphagia; swallowing impairment is not the leading cause of severe loss of appetite.

In general, the CNAQ was not validated for ALS or other neurological disorders, however, we have chosen this assessment tool because of the absence of motor items. During the past several years, the interest in patient reported outcomes (PRO) has increased. The US Food and Drug Administration released different recommendations for the use of PRO in order to measure the health status, the quality of life, or the evaluation of treatments. There is a need for computer-based symptom related self-assessment from the patients’ perspective in order to optimize the treatment, to support the caregivers, and maintain the quality of life in patients better than using surrogate markers. To improve compliance and acceptance in patients, the use of an online self-assessment tool at home in a calm setting may help facilitate communication between the clinicians and their patients. Especially for immobilized patients with chronic diseases or patients in palliative care, an online tool for measuring symptoms and reporting PROs are useful tools for future treatments and studies. Further advantages of an online assessing tool are reliable storage, ubiquitous access, fast transmission, and immediate processing of data. In the sense of already established telemedicine and future infrastructural developments, it would be desirable to have a live interaction between patients and clinicians, with the possibility that clinicians could respond to critical patient information instantly via the Internet.

Our findings correspond with the clinical experience that many patients present with unintentional weight loss and a declining nutritional status, independent of dysphagia. Muscle wasting and cachexia may occur in the early course of ALS, without the presence of bulbar symptoms. Dysphagia was replaced by severe loss of appetite as the independent risk factor for unintended weight loss in ALS. The cause of appetite loss in ALS is not completely understood. Previous studies proposed a correlation between resting energy expenditure and respiratory function [24,25]. In fact, we found a significant association between dyspnea and loss of appetite. Loss of appetite occurred more often in patients with dyspnea compared to patients without dyspnea. Our observations suggested that increased respiratory effort promotes a loss of appetite. This result may be explained by early satiety after eating small amounts due to ALS-related weakness of the patients’ diaphragm [26], supported with evidence from patients with paralysis of the diaphragm who developed peri- or postprandial dyspnea and fatigue [26]. More speculatively, the known change of inflammatory status related to respiratory failure may reduce appetite [12,27-30].

The observed effect of respiratory disturbances is unlikely to be related solely to modifications of patients´ diet due to bulbar dysfunction, since dysphagia was not a risk factor for loss of appetite. In our study, 12 patients using NIV were enrolled. Severe loss of appetite occurred less frequently in the NIV group (42%, 5/12) as compared to patients without NIV (49%, 19/39). Although it is well-known that NIV may reduce energy expenditure and prevent negative effects of dyspnea on satiety, the data of our study did not reach statistical significance and was limited by small sample numbers. However this might be an area worthy of development alongside studies of NIV effectiveness.

### Limitations and Further Research

Limitations of the current study included recruitment of patients from a single specialist ALS center, a relatively small sample size (particularly for subgroup analysis), and the absence of detailed dietary or metabolic assessments. Despite the fact that the CNAQ has not been validated in the context of ALS, our results point towards the same direction as the prediction of at least 5% weight loss within 6 months [14]. For validation of the CNAQ within ALS, it would be necessary to examine the quality criteria objectivity, reliability, and validity. It would also be essential to standardize the CNAQ-based results in a representative cohort of ALS patients and to compare them with an equivalent assessment tool. Furthermore, the results of the validation of the CNAQ in ALS patients should be compared with those of the applied CNAQ in long-term care in institutionalized and community-dwelling adults. Additional weaknesses of the paper are the missing assessment of depression as one reason for appetite loss as well as possible cognitive impairments regarding answering the relevant questionnaires during our investigation. These should be addressed in further trials investigating loss of appetite.

However, the results of the study had benefitted from a longitudinal time course, enabled in part by the novel use of an online patient portal to collect clinically validated health data. Such systems have the potential to accelerate clinical research in ALS, whether fielded in the context of clinical management (such as ALSHome. [17]) or an independent platform such as PatientsLikeMe [31,32] because once the infrastructure is in place, there is little or no incremental cost for fielding research surveys, which patients can do at home and in their own time.

Because the etiologies of severe loss of appetite are heterogeneous, several approaches to treatment of reduced appetite have been reported. However, most of the studies have been performed in the context of malnutrition from cachexia in patients with cancer [33]. Pharmacological agents have been investigated in an attempt to favorably affect appetite including progestagens, corticosteroids, cannabinoids, olanzapine, and mirtazapine [33,34]. In ALS, these agents have been rarely used. There are still many questions with regard to the implication of severe loss of appetite and its direct effect on nutritional status, survival, or most importantly, quality of life. Given the open questions, the impact of early satiety and reduced appetite has to be investigated in larger studies. From these studies, we will conclude whether interventions such as appetite-stimulating pharmacotherapy are justified and potentially successful. The timely detection and treatment of loss of appetite may contribute to improved palliation for patients with ALS.

## Abbreviations

ALS: amyotrophic lateral sclerosis
ALSFRS-R: amyotrophic lateral sclerosis functional rating scale revised
BMI: body mass index
CNAQ: Council on Nutrition Appetite Questionnaire
NIV: non-invasive ventilation
PEG: percutaneous endoscopic gastrostomy
PRO: patient reported outcomes
